# Longitudinal real-world surveillance of infection outcomes in CAR-T and bispecific therapy recipients: the CLARITY study protocol

**DOI:** 10.1136/bmjopen-2025-111194

**Published:** 2026-05-07

**Authors:** Gemma K Reynolds, Mary Ann Anderson, Karin Thursky, Benjamin W Teh, Monica A Slavin

**Affiliations:** 1 Department of Infectious Diseases, Peter MacCallum Cancer Centre, Melbourne, Victoria, Australia; 2 Sir Peter MacCallum Department of Oncology, University of Melbourne, Parkville, Victoria, Australia; 3 National Centre for Infections in Cancer, Peter MacCallum Cancer Centre, Melbourne, Victoria, Australia; 4 Department of Infectious Diseases and Immunology, Austin Health, Melbourne, Victoria, Australia; 5 Department of Clinical Haematology, Peter MacCallum Cancer Centre and Royal Melbourne Hospital, Melbourne, Victoria, Australia; 6 Division of Blood Cells and Blood Cancer, Walter and Eliza Hall Institute Melbourne, Melbourne, Victoria, Australia

**Keywords:** Infection control, HAEMATOLOGY, Epidemiology, Lymphoma

## Abstract

**Abstract:**

**Introduction:**

Infections are a leading cause of non-relapse mortality following chimeric antigen receptor T-cell therapy (CAR-T) and bispecific antibody (BsAb) therapies. However, infection data from clinical trials are often incomplete, lack pathogen-level detail and rarely capture late infectious complications. This **C**AR-T treatment in **L**ymphoma: **A**nalysis of **R**isk of **I**nfection following **T**herap**y** (CLARITY) study aims to generate real-world, longitudinal infection data with extended follow-up to characterise infection timing, including late events and inform risk prediction in patients with lymphoma and myeloma receiving novel immunotherapies.

**Methods and analysis:**

CLARITY is a multicentre observational cohort study across six Australian centres enrolling adults treated with CAR-T or BsAb therapies. A co-designed REDCap (Research Electronic Data Capture) instrument captures infections classified as microbiologically defined, clinically defined or fever of unknown origin, using internationally standardised definitions. Patients were enrolled between 2019 and 2023, with at least 2 years follow-up per patient, allowing time-updated data on immunosuppressive exposures, haematological recovery and prophylaxis. Multivariable regression and landmark analyses will estimate infection incidence and identify dynamic risk factors over time. Incidence rate ratios will assess prophylaxis effectiveness. Data integrity is supported by central adjudication and site-level audits.

**Ethics and dissemination:**

The study has received a waiver of consent (HREC/PMCC/89002) and was co-designed by haematology and infectious diseases investigators. Findings will be disseminated through peer-reviewed publications, scientific meetings and national guideline committees to inform infection prevention and late effects surveillance in immunotherapy-treated populations.

**STRENGTHS AND LIMITATIONS OF THIS STUDY**

- A longitudinal, real-world cohort design enabled capture of late infections and immune recovery beyond standard clinical trial follow-up.
- A national multicentre approach allowed assessment of geographically relevant infection patterns and benchmarking of institutional prophylaxis practices.
- Internationally standardised infection definitions (microbiologically defined infection, clinically defined infection and fever of unknown origin) were applied to ensure consistency and comparability across sites and studies.
- Time-updated statistical modelling incorporated dynamic clinical exposures, including immunosuppression, haematological recovery and antimicrobial prophylaxis.
- Methodological limitations include potential selection and reporting biases from voluntary case capture, exclusion of clinical-trial patients and protocol-dependent infection surveillance.

## Background

 Infections are the leading cause of non-relapse mortality in patients receiving chimeric antigen receptor T-cell (CAR-T) or bispecific antibody (BsAb) therapy for lymphoma and myeloma.^1 2^ Despite this, comprehensive reporting of infections in clinical trials is limited by incomplete data, lack of standardised definitions and short follow-up durations for safety outcomes.^3 4^

Systematic reviews have highlighted consistent under-reporting of infection outcomes in patients treated with cellular therapies. In an early meta-analysis of 45 CAR-T studies, 73% reported any-grade infection, while 31% reported only severe infections.^5^ Just 60% of studies reported infections by causative pathogen.^5^ In a more recent meta-analysis, the cause of fatal infections in patients treated on CAR-T trial for lymphoma was unascertainable in two-thirds of cases.^1^ Similarly, in BsAb-treated lymphoma patients, only 42% of grade ≥3 infections had a reported aetiology.^6^ With the rapid regulatory approval of these therapies,^7^,^9^ robust infection data are essential to protect patients entering routine care.

Trial-based reporting often lacks alignment with international consensus guidelines for immunocompromised populations.^4 10^ Adverse events are typically reported using CTCAE V.5, which categorises infections by anatomical site (eg, “skin infection”) and includes a limited list of pathogens without syndrome-level classification. This limits the ability to aggregate pathogen data and detect early opportunistic infection signals. Adopting peer-reviewed classification systems^4^ and defining microbiologically confirmed, clinically defined or fever of unknown origin (FUO) can improve reporting consistency and clinical interpretability.^10^

Short follow-up and inconsistent prophylaxis reporting further hinder risk characterisation. Early-phase CAR-T trials typically report outcomes for only 6 months,^5 6^ prompting calls for longer-term studies.^11 12^ Large databases often censor at day 100,^13^ even when therapy occurred years prior. Institution-specific prophylaxis strategies are rarely published. Furthermore, the early reliance on pooled proportions, rather than incidence rates, limits the ability to define periods of infection risk.^14 15^

Accurate understanding of infection risk also requires data on treatment-emergent adverse events and immunosuppressive exposures. Cytokine release syndrome (CRS) and immune-effector cell associated neurotoxicity (ICANS) are strongly associated with infection^16 17^ and are treated with agents that further compound immunosuppression including high dose glucocorticoids and tocilizumab. Despite this, cumulative exposures to additional immunosuppressive agents are not systematically captured in many datasets.^18^ A standardised reporting approach is needed.

While infection outcomes following CAR-T therapy are well understood within individual Australian centres, national-level epidemiologic data are lacking. This study aims to provide descriptive insight into infection patterns in this specialised population, with sufficient power to explore risk prediction.

## Study objectives

The primary objectives of the CLARITY study (**C**AR-T treatment in **L**ymphoma: **A**nalysis of **R**isk of **I**nfection following **T**herap**y**) are to:

1. Determine the incidence of infections in patients with lymphoproliferative neoplasms treated with CAR-T or BsAb therapies.
2. Determine the incidence of microbiologically defined infections (MDIs)—defined as infection syndromes with a corresponding positive microbiological result.
3. Determine the incidence of clinically defined infections (CDIs)—defined as infection syndromes without a positive microbiological result.
4. Determine the incidence of FUO—including:
  - FUO likely attributable to CRS.
  - FUO without a known aetiology.
5. Assess the grade and severity of infections using standardised criteria.

Secondary objectives include:

1. Describe prophylaxis strategies (antibacterial, antifungal and antiviral) used in clinical care.
2. Evaluate the association between infection risk and key clinical factors, including:
  - CRS and ICANS.
  - Haematotoxicity: neutropenia, hypogammaglobulinaemia and lymphopenia.
  - Use of antimicrobial prophylaxis.

- CRS and ICANS.
- Haematotoxicity: neutropenia, hypogammaglobulinaemia and lymphopenia.
- Use of antimicrobial prophylaxis.

The protocol is reported according to Strengthening the Reporting of Observational Studies in Epidemiology checklist for Observational Cohort studies (see online supplemental).

## Materials and methods

### Study design and setting

This is a national multicentre cohort study designed to support dynamic site inclusion, expansion across therapeutic platforms, and longitudinal assessment of infection outcomes and risk factors in patients treated with CAR-T cell therapies and bispecific antibodies. This study recruited patients treated with CAR-T between January 2019 and December 2023, with long-term follow-up and data censor planned in late 2025.

### Hospital selection

The study includes the six adult Australian centres currently providing standard-of-care CAR-T therapy for lymphoma. Additional sites may be included as new therapies become available or as institutional capabilities expand.

### Selection of cellular therapies

The study includes standard-of-care CD19-directed CAR-T products (tisagenlecleucel and axicabtagene). When BCMA-directed CAR-T becomes standard-of-care for myeloma, it will be incorporated under the existing ethics approval. For BsAb therapies, patient inclusion is subject to institutional agreements aligned with regulatory and contractual obligations. As these therapies are primarily available through clinical trials or compassionate access programmes, data collection will occur only with appropriate institutional approvals and sponsor agreement. Should bispecific therapies become standard-of-care during the study period, data sharing and inclusion processes will be revised accordingly to ensure governance and ethical compliance.

### Patient selection

Inclusion criteria:

1. Age 18 years or older.
2. Histological diagnosis of a lymphoproliferative neoplasm (including acute leukaemia, lymphoma, myeloma or chronic lymphocytic leukaemia).
3. Medical records available for clinical review.
4. Treated with the intent of achieving disease remission.
5. Therapy received under compassionate access, standard-of-care or within a clinical trial.

The study focuses on infection outcomes following standard-of-care CAR-T or bispecific therapy, as defined in the study case report forms. For patients treated in clinical trials, inclusion will be subject to approval by the trial principal investigator and other institutional stakeholders. The study will not identify individual trial names, investigational products or commercial therapies in its analysis or dissemination.

To facilitate assessment of enrolment bias, the primary cellular therapies sites will contribute further denominator data by providing the number of patients treated with cellular therapies, but who were excluded from this study, and the reason for exclusion (eg, age, trial participant).

### Patient and public involvement

Three consumer representatives were involved at the concept development stage, where lived experience informed the focus on late infections beyond routine trial follow-up, particularly vaccine-preventable and respiratory viral infections. Their input helped shape the research questions and prioritised clinically meaningful outcomes related to long-term infectious morbidity. Consumers provided feedback on study design and the types of questions asked of the data but were not involved in recruitment, data collection or protocol drafting for this retrospective cohort study. There was no perceived participant burden, as all data were derived from routinely collected medical records, although consumer contributors were compensated for their time reviewing the methodology. Consumer involvement in dissemination is planned once results are available, including guidance on how findings are shared with patient and community audiences.

### Data collection and management

This study recruited patients treated with CAR-T between January 2019 and December 2023. Recruitment commenced in 2024, with ongoing patient inclusion and longitudinal follow-up focused on late infections, with data censoring planned in late 2025 to provide opportunity for at least 2 years of follow-up per patient. Data are being collected using REDCap, a secure web-based platform for research data management. As follow-up is longitudinal, data collection remains active and is not yet complete.

Each participating site has appointed a principal investigator with relevant clinical and research experience to oversee protocol adherence and local data entry. While site teams vary in composition, inclusion of an antimicrobial stewardship pharmacist is encouraged to support case identification and data completeness. All study personnel receive training in medical record review, infection classification and REDCap use. Oversight is provided by the Chief and Coordinating Principal Investigators.

The study captures patient-level data, infection outcomes including healthcare-associated and opportunistic infections, and site-level infection prevention practices. A centralised data management strategy ensures secure and accurate handling of all study information.

Often, patients receiving CAR-T are discharged back from the cellular therapies centre to their referring haematology centre at 1-year post follow-up. Two strategies will be employed to capture late infection data. Where possible, high-volume haematology centres who refer frequently to CAR-T will also be recruited as part of the study to provide long-term infection outcomes from the referring site. Alternatively, correspondence between referring haematology centres and cellular therapies centres will be available for viewing under the medical record of the cellular therapy centre. There is not a national electronic medical record system available in Australia to support this study.

### Patient data

Patient-level data will be extracted from the medical record to record demographics, infectious events, and relevant haematological and treatment characteristics (see table 1). These will be used to assess infection risk factors and clinical outcomes.

**Table 1:** Patient data for CAR-T treatment in Lymphoma: Analysis of Risk of Infection following Therapy study

Patient demographics	Age
Haematological malignancy	Year of diagnosis Prior treatments Prior autologous or allogeneic stem cell transplant
CAR-T treatment	CAR-T product CAR-T conditioning
Infection events (see text)	Microbiologically-defined infections (bacterial, viral, fungal and parasitic) Antimicrobial susceptibility for identified pathogens Clinically-defined infections Fever of unknown origin Neutropenic fever Infection grading Infection outcomes
Risk factors for infection (see text)	Baseline CAR-HEMATOTOX score^26 28^ Steroid and tocilizumab exposure in first 28-days post CAR-T Steroid exposure in 14- and 60-days prior to infection episode Neutrophil recovery following CAR-T (monophasic, biphasic and aplastic; and immune-cell associated haematotoxicity definitions)^26^ Hypogammaglobulinaemia and IVIG replacement Lymphocyte recovery (absolute lymphocyte count, CD3+ and CD4+)

CAR-T, chimeric antigen receptor T-cell; IVIG, intravenous immunoglobulin.

### Infection definitions

Infection events will be classified according to international consensus guidelines for reporting infections in immunocompromised hosts,^4^ using bespoke REDCap instrument (online supplemental 1). Each event will be categorised by the treating centre as one of the following:

- MDI: an infection syndrome with a corresponding positive microbiological result. For each MDI, data on causative pathogen, infection site and antimicrobial susceptibility will be collected.
- CDI: an infection syndrome without a microbiological result. Data will include the suspected clinical site and likely pathogen category.
- FUO: febrile episodes without a confirmed infection or alternative explanation. These will be subclassified as:
  - FUO likely attributable to CRS.
  - FUO without evidence of CRS or other defined cause.

Targeted data will be collected for key opportunistic infections including cytomegalovirus (CMV), COVID-19 and invasive fungal infections. For each, treatment and outcome data will be recorded. Reporting will align with updated international definitions (such as European Organisation for Research and Treatment of Cancer/Mycoses Study Group [EORTC/MSG] definitions of invasive fungal infection) to enable cross-study comparisons and standardisation.^19 20^

Infection timing will be analysed. Early infections will be defined as infections between CAR-T infusion (day 0) and day 30 post CAR-T, to be consistent with time periods of previous studies.^21^ Infections occurring within the first year, between day 31 and 365 will be analysed for generalisability with earlier studies. Late infections, occurring more than 365 days after CAR-T infusions, will also be collected.^22^,^24^

Infection-related morbidity and mortality will be captured using CTCAE severity grading, site-adjudicated attribution of cause of death, timing of infection relative to therapy and all-cause mortality. This consistent approach will support detailed analyses of infection burden and risk in patients receiving CAR-T or bispecific therapies.

### Risk factors for infection

This study will support time-updated analyses of infection risk based on treatment exposures, immune reconstitution and baseline clinical characteristics.

Treatment-emergent complications such as CRS, ICANS, neutropenia and lymphopenia will be recorded both during the initial admission (through day 28) and in proximity to any infection events. These events will be graded using standard consensus criteria,^25^ and accepted as documented by the treating haematology team without re-adjudication.

Glucocorticoid exposure will be recorded as cumulative dexamethasone-equivalent dose (in milligrams) over three specific time windows: (a) Within 28 days after therapy commencement, (b) Within 14 days prior to infection and (c) Within 60 days prior to infection. Tocilizumab exposure in the 28 days before infection will also be recorded.

Haematological recovery will be assessed both qualitatively (as monophasic, biphasic or aplastic patterns)^26^ and quantitatively. Absolute neutrophil nadir will be assessed within 14 days before each infection event. Lymphocyte recovery, including total lymphocyte count, CD3+ and CD4+ levels, will be evaluated within 90 days before each infection. Hypogammaglobulinaemia will be recorded as a diagnosis, with or without active intravenous immunoglobulin (IVIG) replacement. This structured framework will enable analysis of dynamic infection risk in relation to immune recovery, treatment intensity and prophylaxis use.

### Prophylaxis

Antiviral, antifungal, anti-Pneumocystis and antibacterial prophylaxis will be documented at three time points: (a) Immediately following therapy infusion, (b) At last clinical follow-up and (c) At the time of any infection event. The specific agents used and changes over time will be recorded to support time-dependent analyses of prophylaxis effectiveness.

### Study size

The CLARITY study aims to improve our understanding of real-world infections in patients treated with cellular therapies. As this treatment modality is new, its utilisation and clinical application are rapidly expanding, with both more patients being treated at established centres and more centres becoming available for treatment. It is estimated that by the end of 2023, 350 patients were treated as standard of care in Australia on CAR-T, with 80% recruitment of participants we anticipate a cohort of 280 patients.

### Analysis

Analyses will be undertaken with a statistician and assess both infection incidence and the association between predefined clinical risk factors and infection outcomes.

Descriptive statistics will summarise baseline patient characteristics, prophylaxis strategies and infection events. The cumulative incidence of infection overall and by subtype (microbiologically defined, clinically defined, FUO) will be estimated. Planned sensitivity analysis of infection incidence will be undertaken by CAR-T product (axicabtagene vs tisagenlecleucel) due to established differences in infection incidence,^17^ and by underlying haematological malignancy.^5^

Multivariable regression models will be used to examine associations between infection risk and clinical variables, with clinical variables selected based on previous studies.^16^ Time-updated covariates will allow assessment of the impact of CRS, neutropenia, lymphopenia, hypogammaglobulinaemia and prophylaxis use. Backward stepwise regression with a p value cut-off of <0.2 will be used. Infection prediction models will be created for (a) All-grade infections, (b) Severe grade ≥3 infections, (c) MDIs and (d) Bacterial, viral and fungal infections. Sensitivity analyses will be undertaken using landmark analyses for risk of infection during early, intermediate and late time periods. Cox proportional hazards models will then be used to analyse risks associated with first bacterial, viral and fungal infection. Threshold analyses will be conducted to explore exposure–response relationships for fungal and viral infections and identify clinically relevant risk thresholds.

For prophylaxis that mirrors change in haematoxicity, for example, re-initiation for mould-active prophylaxis for longstanding neutropenia or IVIG for hypogammaglobulinaemia, the effectiveness of prophylaxis strategies will be assessed using incidence rate ratios (IRRs). IRRs will compare periods with and without prophylaxis in time-dependent models. For prophylaxis that is used for a fixed duration, for example, anti-viral therapy for 12-months following CAR-T, breakthrough infections will be evaluated as a marker of prophylactic efficacy. All analyses will account for time at risk and adjust for potential confounders specified a priori. Sensitivity analyses will address the impact of missing data and variability in infection definitions.

## Data management

### Data monitoring and validation

Data collection and monitoring will follow principles of good clinical practice and institutional governance. Each site will ensure accurate and complete data entry under the oversight of a local principal investigator. Clinical events including infections, adverse outcomes and treatment exposures will undergo multidisciplinary review by haematology and infectious diseases teams where feasible.

A central study team will conduct periodic data audits, resolve queries and review key outcomes such as infection classification and cause of death. Complex or unclear cases will be referred to a central adjudication panel composed of infectious diseases physicians, haematologists and study investigators. Data completeness and accuracy will be reviewed regularly by the Chief and Coordinating Principal Investigators.

### Data storage and preservation

Access to the database is restricted to authorised individuals. At each participating site, data collection will be undertaken by the clinical research team under the oversight of the site’s principal investigator. All study data will be maintained on a secure, encrypted server with access limited to the study investigators.

### Data analysis

During the analysis phase, investigators from participating institutions will access only deidentified data relevant to the study aims. All analyses will be performed in line with the prespecified study methods to ensure accuracy and reliability. No individual participant will be identifiable in any reports, presentations or publications arising from this project.

### Data governance

The Principal Investigator will serve as the REDCap Data Custodian, with responsibility for overseeing the collection and management of study data. In this role, the investigator is also accountable for the analysis, long-term stewardship and preservation of the dataset through to dissemination of study findings.

### Data sharing

Study sites may share deidentified patient data with external collaborators from other institutions, provided written approval is obtained from the lead investigator and only for participants who have consented to future research use. Prior to any transfer, a formal data sharing agreement will be established between the study site and collaborating institution. Such agreements will explicitly safeguard data security and uphold patient confidentiality.

### Participant consent

This study has been granted a waiver of consent by the Human Research Ethics Committee (HREC/PMCC/89002), in accordance with national guidelines for low-risk research using retrospective or routinely collected health data.

### Dissemination

Study findings will be shared through presentations at national and international meetings, peer-reviewed publications and collaboration with professional societies. Where applicable, results will be shared with clinical guideline committees and national working groups to inform evidence-based recommendations. The study aims to support both clinical practice and policy through high-quality, real-world evidence on infection outcomes in novel immunotherapy recipients.

### Ethics and co-design

The infection and data collection framework were co-designed by infectious diseases physicians and haematologists, all of whom are listed as study investigators. Data collection commenced for patients receiving standard-of-care CAR-T therapy for lymphoma between 2019 and 2023 under HREC/PMCC/89002. Participant entry will be censored at the end of 2023, with ongoing follow-up through to late 2025 to capture longitudinal outcomes and late infections.

### Discussion

Understanding infection risk in patients receiving novel immunotherapies is a dynamic challenge that evolves in tandem with rapid therapeutic advancement. The primary strength of this study is its duration of planned follow-up and aim to capture late infections, which remains a significant clinical and research priority.^11^ Other strengths include standardised definitions of infections, which will ensure comparability across sites and over time. It also systematically captures immunosuppressive exposures including corticosteroids and cytokine-directed agents to allow more granular exploration and attribution of infection risk.

To future-proof this study and maximise its impact, following study completion, the data will be linked to an existing, prospective national CAR-T database. Incorporating retrospectively collected data into the prospective protocol will allow future infection outcomes to be linked with treatment-related toxicities and subsequent treatment refined during the enrolment period of this study. Importantly, the retrospective phase will also function as a feasibility assessment of the data collection instrument from the existing medical record, enabling evaluation of data quality, completeness and operational burden prior to its use for sustained prospective collection. This approach may also reduce bias associated with voluntary data capture by streamlining and reconciling the number of competing studies requiring participation.

**Figure 1:**
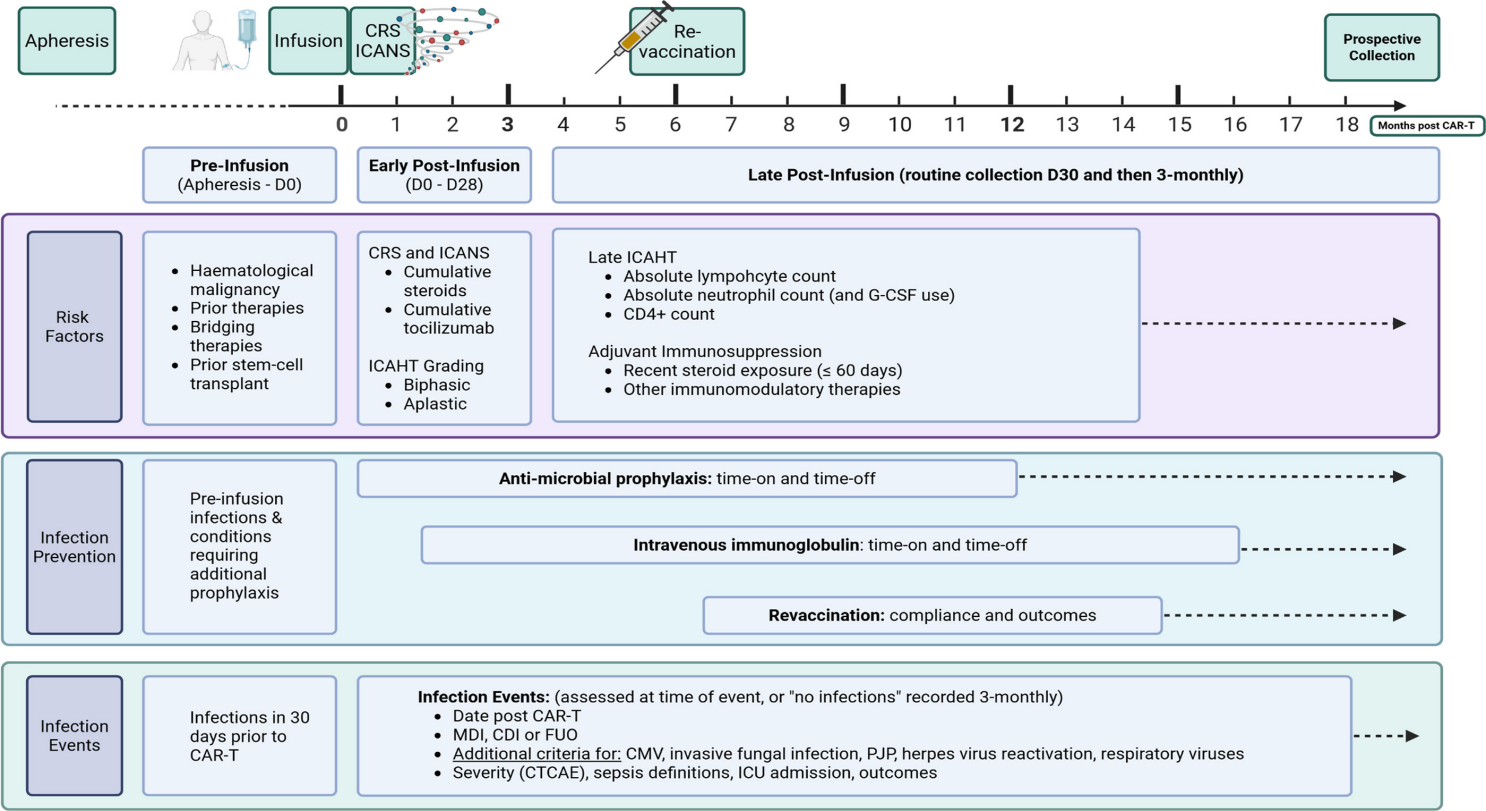
Conceptualisation of data collection for infection risks in patients treated with cellular therapies. CAR-T, chimeric antigen receptor T-cell; CDI, clinically defined infection; CRS, cytokine release syndrome; CMV, cytomegalovirus; FUO, fever of unknown origin; ICU, intensive care unit; ICAHT, immune cell associated haematotoxicity; ICANS, immune-cell associated neurotoxicity; MDI, microbiologically defined infection; PJP, pneumocystis pneumonia.^30^

For example, the consensus statement defining and standardising reporting for immune effector-cell associated haematotoxicity was finalised in September 2023^27^ and thus may not have been routinely collected by centres for patients enrolled from earlier treatment time points. Similarly, the role of the CAR-HEMATOTOX score,^26^ first published in association with infection following CAR-T for lymphoma in 2022,^28^ is increasingly appreciated as a risk for infection but may not have been collected at earlier enrolment time points. Screening practices for opportunistic infections, particularly CMV, have also evolved significantly over the study period defined in this protocol.^29^ National linkage facilitates incorporation of consensus up-to-date measures of treatment-associated toxicity, supportive care practices and haematological outcomes, while maintaining comparability over time via a versioned data dictionary and ability to undertake backwards-mapping of legacy variables (figure 1). This will ensure the most accurate attribution of infection risk and enables timely benchmarking across centres, products and timing of cellular therapy treatment.

## Supplementary material

10.1136/bmjopen-2025-111194 online supplemental file 1

10.1136/bmjopen-2025-111194 online supplemental file 2

## References

[1] Cordas Dos Santos DM, Tix T, Shouval R (2024). A systematic review and meta-analysis of nonrelapse mortality after CAR T cell therapy. Nat Med.

[2] Tix T, Alhomoud M, Shouval R (2025). Non-relapse mortality with bispecific antibodies: a systematic review and meta-analysis in lymphoma and multiple myeloma. Mol Ther.

[3] Cliff ERS, Reynolds G, Popat R (2023). Acknowledging Infection Risk in Bispecific Antibody Trials in the Treatment of Multiple Myeloma. J Clin Oncol.

[4] Teh BW, Mikulska M, Averbuch D (2024). Consensus position statement on advancing the standardised reporting of infection events in immunocompromised patients. Lancet Infect Dis.

[5] Reynolds GK, Sim B, Spelman T (2023). Infections in haematology patients treated with CAR-T therapies: a systematic review and meta-analysis. Crit Rev Oncol Hematol.

[6] Reynolds GK, Maclean M, Cliff ERS (2024). Infections in patients with lymphoma treated with bispecific antibodies: a systematic review and meta-analysis. *Blood Adv*.

[7] Cliff ERS, Rome RS, Kesselheim AS (2023). National Comprehensive Cancer Network Guideline Recommendations of Cancer Drugs With Accelerated Approval. JAMA Netw Open.

[8] Liu ITT, Kesselheim AS, Cliff ERS (2024). Clinical Benefit and Regulatory Outcomes of Cancer Drugs Receiving Accelerated Approval. JAMA.

[9] Ge AY, Feldman WB, Kaiser MF (2024). Global Access to Chimeric Antigen Receptor (CAR) T-Cell Therapies: Health Technology Assessment (HTA) of CAR T in G7 Countries and Australia. Blood.

[10] Teh BW, Reynolds GK, Mikulska M (2024). Improving infection reporting in hematology treatment trials. *Blood Adv*.

[11] Shahid Z, Jain T, Dioverti V (2024). Best practice considerations by the american society of transplant and cellular therapy: infection prevention and management after chimeric antigen receptor T cell therapy for hematological malignancies. Transplant Cell Ther.

[12] Kampouri E, Little JS, Rejeski K (2023). Infections after chimeric antigen receptor (CAR)-T-cell therapy for hematologic malignancies. *Transpl Infect Dis*.

[13] Wudhikarn K, Herr MM, Chen M (2025). Infection after CD19 chimeric antigen receptor T-cell therapy for large B-cell lymphoma: real-world analysis from CIBMTR. Blood Adv.

[14] Ludwig H, Munshi NC, Terpos E (2024). Proposal for harmonizing the reporting of infections during treatment with bispecific antibodies in multiple myeloma. Blood Adv.

[15] van Besien H, Easwar N, Demetres M (2025). Comparative infection risk in CAR T vs bispecific antibodies in B-cell lymphoma: a systematic review and meta-analysis. Blood Adv.

[16] Reynolds G, Sim B, Anderson MA (2023). Predicting infections in patients with haematological malignancies treated with chimeric antigen receptor T-cell therapies: a systematic scoping review and narrative synthesis. Clin Microbiol Infect.

[17] Hill JA, Seo SK (2020). How I prevent infections in patients receiving CD19-targeted chimeric antigen receptor T cells for B-cell malignancies. Blood.

[18] Sassine J, Agudelo Higuita NI, Siegrist EA (2024). Timeline and outcomes of viral and fungal infections after chimeric antigen receptor T-cell therapy: a large database analysis. Clin Microbiol Infect.

[19] Donnelly JP, Chen SC, Kauffman CA (2019). Revision and update of the consensus definitions of invasive fungal disease from the european organization for research and treatment of cancer and the mycoses study group education and research consortium. Clin Infect Dis.

[20] Ljungman P, Chemaly RF, Khawaya F (2024). Consensus Definitions of Cytomegalovirus (CMV) Infection and Disease in Transplant Patients Including Resistant and Refractory CMV for Use in Clinical Trials: 2024 Update From the Transplant Associated Virus Infections Forum. Clin Infect Dis.

[21] Hill JA, Li D, Hay KA (2018). Infectious complications of CD19-targeted chimeric antigen receptor-modified T-cell immunotherapy. Blood.

[22] Logue JM, Zucchetti E, Bachmeier CA (2021). Immune reconstitution and associated infections following axicabtagene ciloleucel in relapsed or refractory large B-cell lymphoma. Haematologica.

[23] Thakkar A, Cui Z, Peeke SZ (2020). Dynamics of Leukocyte Subpopulations Reconstitution Predict Infection Propensity in a Multiethnic Real World Cohort Treated with Anti-CD19 CAR-T Cell Therapy (Axicabtagene-Ciloleucel). Blood.

[24] Thakkar A, Cui Z, Peeke SZ (2021). Patterns of leukocyte recovery predict infectious complications after CD19 CAR-T cell therapy in a real-world setting. Stem Cell Investig.

[25] Lee DW, Santomasso BD, Locke FL (2019). ASTCT consensus grading for cytokine release syndrome and neurologic toxicity associated with immune effector cells. Biol Blood Marrow Transplant.

[26] Rejeski K, Perez A, Sesques P (2021). CAR-HEMATOTOX: a model for CAR T-cell-related hematologic toxicity in relapsed/refractory large B-cell lymphoma. Blood.

[27] Rejeski K, Subklewe M, Aljurf M (2023). Immune effector cell-associated hematotoxicity: EHA/EBMT consensus grading and best practice recommendations. Blood.

[28] Rejeski K, Perez A, Iacoboni G (2022). The CAR-HEMATOTOX risk-stratifies patients for severe infections and disease progression after CD19 CAR-T in R/R LBCL. J Immunother Cancer.

[29] Kampouri E, Ibrahimi SS, Xie H (2024). Cytomegalovirus (CMV) Reactivation and CMV-Specific Cell-Mediated Immunity After Chimeric Antigen Receptor T-Cell Therapy. Clin Infect Dis.

[30] Kröger N, Gribben J, Chabannon C (2022). The EBMT/EHA CAR-T cell handbook.

