# Supplementary material for: Longitudinal real-world surveillance of infection outcomes in CAR-T and bispecific therapy recipients: the CLARITY study protocol
Source: BMJ Open. 2026 May 7;16(5):e111194. doi: 10.1136/bmjopen-2025-111194 (PMC13157767; doi:10.1136/bmjopen-2025-111194)
Supplement: online supplemental file 1 [file bmjopen-16-5-s001.pdf]

Supplement 1: RedCap Infection Data Collection Instrument

Record ID

____

What is the patient's month and year of birth?

____
(mm/yyyy)

Haematological Treatment History

What is the patient's haematological diagnosis at time of receiving CAR T therapy?

- ◯ Diffuse large B-cell lymphoma (no Richter's transformation)
- ◯ Follicular lymphoma
- ◯ Marginal zone lymphoma
- ◯ Mantle cell lymphoma
- ◯ Acute lymphoblastic leukaemia
- ◯ Multiple myeloma
- ◯ Chronic lymphocytic leukaemia
- ◯ Other
- ◯ Diffuse large B-cell lymphoma (Richter's transformation)

In what year did the initial haematological diagnosis occur?

____

How many lines of therapy has the patient received prior to CAR T-cell therapy?

- ◯ 1
- ◯ 2
- ◯ 3
- ◯ 4
- ◯ 5
- ◯ 6
- ◯ 7
- ◯ 8
- ◯ 9
- ◯ 10 or more
- ◯ 0; bispecific represents firstline therapy

(Number of discrete lines of therapy prescribed. Please inclued consolidation cycles as part of the same treatment line (e.g. 2 x subsequent rituximab following R-CVP corresponds to one line of therapy). Radiation therapy is included as a treatment line. Transplantation (allogeneic/autologous) is included as a treatment line. Do not include the conditioning regimen for CAR-T)

Please provide a summary of the lines of chemotherapy prescribed; dates are not required, abbreviations are accepted.

____
(For example: 6 x R-CHOP, 4 x CVP, 2 x R)

What was the lymphodepletion regimen used prior to CAR T-cell infusion?

- ◯ Fludarabine / Cyclophosphamide
- ◯ Bendamustine
- ◯ Cyclophosphamide
- ◯ Fludarabine
- ◯ Other

Has the patient received a prior stem cell transplant (SCT)?	◯ Yes - autologous SCT<br>◯ Yes - allogeneic SCT<br>◯ No prior SCT<br>(SCT must have been performed prior to the receipt of the bispecific therapy.)
What was the year of stem cell transplant?	______________________________
Has the patient received a previous cellular therapy or other CAR T-cell therapy?	◯ Yes - prior CAR-T<br>◯ Yes - another prior bispecific<br>◯ No - neither cellular nor previous bispecific therapy<br>(Refers to treatment received prior to current bispecific therapy.)
What was the status of the patient's disease at time of receiving CAR T-cell therapy?	◯ Relapsed disease<br>◯ Refractory disease<br>◯ De novo disease<br>◯ Disease in remission<br>◯ Other
What was the stage of the patient's disease at time of receiving the CAR T-cell therapy?	◯ Stage 1<br>◯ Stage 1e<br>◯ Stage 2<br>◯ Stage 2e<br>◯ Stage 2 - bulky disease<br>◯ Stage 3<br>◯ Stage 4
What was the patient's LDH prior to commencement of this CAR T infusion?	
What was the patient's ferritin prior to infusion of the CAR T product?	

Prior to the commencement of CAR T therapy; did the patient of any of the following markers of disease bulk?

	Yes	No
Did the patient have lymph nodes > 5cm but < 10cm?	◯	◯
Did the patient have lymph nodes > 10cm?	◯	◯
Did the patient have bone marrow involvement?	◯	◯
DId the patient have disease above and below the diaphragm?	◯	◯

CAR T therapy

What was the CAR T-cell product received?

- ◯ Abecma (idecabtagene vicleucel)
- ◯ Breyanzi (lisocabtagene maraleucel)
- ◯ Kymriah (tisagenlecleucel)
- ◯ Tecartus (brexucabtagene autoleucel)
- ◯ Yescarta (axicabtagene ciloleucel)
- ◯ Anti-BCMA CAR-T product
- ◯ CAR-NK product

What was the date of CAR T-cell infusion?

____
(dd/mm/yyyy)

What was the date of last haematology follow-up?

____
(dd/mm/yyyy)

At the time of data entry, what is the best description of disease and treatment activity?

- ◯ Complete metabolic remission
- ◯ Partial metabolic remission
- ◯ Progressive disease
- ◯ Death

Did the patient experience neutropenia following CAR-T therapy for > 14 days?

Grade 0 = Neutrophil counts in normal range
Grade 1 = Neutrophils > 1.5 and < 2.0
Grade 2 = Neutrophils > 1.0 and < 1.5
Grade 3 = Neutrophils > 0.5 and < 1.0
Grade 4 = Neutrophils < 0.5

- ◯ No - Grade 0
- ◯ Yes - Grade 1
- ◯ Yes - Grade 2
- ◯ Yes - Grade 3
- ◯ Yes - Grade 4

(Time period from lymphodepletion onwards)

Neutropenia following CAR-T infusion?

	Yes	No
Did the patient develop neutropenia after lymphodepletion, and recover neutrophils (> 1.0) before D28?	◯	◯
Did the patient develop neutropenia after lymphodepletion and fail to count-recover (ANC < 1.0) by D28?	◯	◯
Did the patient develop neutropenia after D28 following a period of initial count recovery	◯	◯

Did the patient experience an episode(s) of cytokine release syndrome following receipt of CAR T-cell therapies?

Please enter the maximum grade experienced if there were multiple episodes.

- ◯ No - Grade 0
- ◯ Yes - Grade 1
- ◯ Yes - Grade 2
- ◯ Yes - Grade 3
- ◯ Yes - Grade 4
- ◯ Yes - Grade 5

(CRS as diagnosed and graded by treating clinician)

Did the patient experience an episode(s) of neurotoxicity / ICANS as part of CAR T-cell treatment?<br><br>Please enter the maximum grade experienced if there were multiple episodes	◯ No - Grade 0<br>◯ Yes - Grade 1<br>◯ Yes - Grade 2<br>◯ Yes - Grade 3<br>◯ Yes - Grade 4<br>◯ Yes - Grade 5<br>(Neurotoxicity or ICANS as diagnosed and graded by treating clinical team.)
Did either CRS or ICANS require the use of dexamethasone and/or tocilizumab as treatment?	◯ Yes - Tocilizumab only<br>◯ Yes - Steroids only<br>◯ Yes - Steroids and Tocilizumab<br>◯ Yes - other treatment (e.g. anakinra)<br>◯ No - did not require treatment
What was the cumulative dose of dexamethasone administered in the 28 days following CAR T therapy?	______________________________<br>(mg)
What was the cumulative dose of tocilizumab (mg) administered in the 28 days following CAR T infusion?	______________________________<br>(mg)
