# Supplementary material for: Longitudinal real-world surveillance of infection outcomes in CAR-T and bispecific therapy recipients: the CLARITY study protocol
Source: BMJ Open. 2026 May 7;16(5):e111194. doi: 10.1136/bmjopen-2025-111194 (PMC13157767; doi:10.1136/bmjopen-2025-111194)
Supplement: Supplementary file 3 [file bmjopen-2025-111194.reviewer_comments.pdf]

PEER REVIEW HISTORY

BMJ Open publishes all reviews undertaken for accepted manuscripts. Reviewers are asked to complete a checklist review form and are provided with free text boxes to elaborate on their assessment. These free text comments are reproduced below.

ARTICLE DETAILS

Title (Provisional)

Longitudinal Real-World Surveillance of Infection Outcomes in CAR-T and Bispecific Therapy Recipients: The CLARITY Study Protocol

Authors

Reynolds, Gemma K; Anderson, Mary A; Thursky, Karin; Teh, Benjamin; Slavin, Monica

---

VERSION 1 - REVIEW

**Reviewer**	**1**
**Name**	**Atkins, Harold**
**Affiliation**	**Ottawa Hospital General Campus, Medicine**
**Date**	**05-Jan-2026**
**COI**	**None**

The authors propose to conduct an observation study of infectious complications of patients that have undergone treatment with CD19-directed CART cells using a framework structure that will allow the inclusion of patients receiving BCMA-directed CART cell therapy or bispecific antibody therapy in the future. The study will use standardized definitions for infectious complications. Incidence of infection during follow-up periods will be measured. Important risk factors for infections will be identified using statistical analyses.

The study protocol is well written and the approach is sound. The data collected will be important to the field, helping to better understand the infectious risks of targeted anticancer cell therapy. Addressing a few minor issues would provide the reader with a better understanding of potential strengths and limitations of the study. These include:

1) It is stated that not all patients that have received CART cells during the enrollment period will be included (for instance, there may be contractual or regulatory barriers to inclusion of patients on certain trials). Collecting and proposing to report the number of excluded patients and the reason for their exclusion (ie trial, etc) would help the reader assess any potential enrollment bias in the data analysis.

2) It is not clear if some of the data has been collected prospectively in a national database. Other data will be collected retrospectively from medical records. The former would presumably be collected in a systematic manner while the later might be less complete, or may need greater interpretation from notes in the medical record. It would strengthen the reader's understanding of the proposed analyses if explicit description of which data has been collected prospectively and which will be collected retrospectively is provided in the protocol

3) CART therapy is often administered at a referral hospital, sometime distant from the patient's home and ongoing health care. Late infectious events and complications might not occur or be documented at the treating hospital. The study protocol should describe if the review of medical records will just be from those available at the treating hospital or will records will be sought from other healthcare institutions. If the study will access medical records through a national electronic medical record system, the protocol should include a description of the systems from which retrospective data will be collected.

4) the study proposes to determine the effectiveness of infection prophylaxis strategies by comparing periods where prophylaxis is given with periods were prophylaxis is not given. However standard practice would use infection prophylaxis during high-risk periods and discontinue the medication during low-risk periods. Thus the proposed comparison is unlikely to provide interpretable information about the effectiveness of prophylaxis. Framing the research question differently (ie - what infections breakthrough prophylaxis) would strengthen the proposed analysis.

VERSION 1 - AUTHOR RESPONSE

**Reviewer Comments**	
**Comment**	**Response**
It is stated that not all patients that have received CAR-T cells during the study period will be included... Collecting and proposing to report the number of excluded patients would help the reader.	Thank you for the suggestion regarding additional denominator data; this is now captured in **lines 162 – 164.**
It is not clear if some data has been collected prospectively in a national database. Other data will be collected retrospectively from the	We agree this is a very important distinction and we have clarified further in text. Please see revised discussion section **lines 345 – 356.**

medial records… It would strengthen the reader's understanding of the proposed analyses if explicit description of which data has been collected prospectively and which will be collected retrospectively is provided in the protocol.	The retrospective study has the advantages of a) dutiful collection of detailed infection data, b) feasibility assessment of the data collection instrument and c) engagement of primary sites.<br><br>Once complete, this data can be linked to a national registry that is currently collecting haematology information, but not detailed infection outcomes. Furthermore, following extensive feasibility assessment of the infection data collection tool across the major centres, it can be adjusted or directly incorporated into the national registry for ongoing data collection by the sites.<br><br>We feel this has two advantages: a) a pilot of data collection instruments to ensure sustainability and longevity, and b) a way to link detailed infection data to toxicity definitions if they change or are re-defined during the study period.
CAR-T therapy is often administered at a referral hospital, sometime distant from the patient's home and ongoing health care. Late infectious events and complications might not occur or be documented at the treating hospital. The study protocols should describe if the review of	This is now clarified in the section "Data Collection and Management" **lines 192 – 198** and outlines either a) enrolment of high-volume referring sites that frequently sent patients for CAR-T and b) the use of correspondence between referring sies and cellular therapy centres ongoing as part of

medical records will just be from those available at the treating hospital, or will records be sought from other healthcare institutions.	the medical record from the cellular therapies centre.
The study proposes to determine the effectiveness of infection prophylaxis strategies by comparing periods where prophylaxis is given with periods where prophylaxis is not given. However, standard practice would use infection prophylaxis during high-risk periods and discontinue the medication during low-risk periods. Thus, the proposed comparison is unlikely to provide interpretable information about the effectiveness of prophylaxis. Framing the research question differently (i.e. what infections breakthrough prophylaxis) would strengthen the proposed analysis.	We thank you for this suggestion and have incorporated additional description in the "Analysis" section (**line 280 – 287**).<br><br>Breakthrough infections will be detailed. Additionally, further detail was given around the types of prophylaxis that are likely to stop and start, particularly mold-active prophylaxis in relation to neutrophils (which can initially recover and then dip with haematotoxicity) as well as intravenous immunoglobulin.

VERSION 2 - REVIEW

**Reviewer**	**1**
**Name**	**Atkins, Harold**
**Affiliation**	**Ottawa Hospital General Campus, Medicine**
**Date**	**06-Mar-2026**
**COI**	

The reply to my previous comments and revisions to the manuscript are satisfactory. I have no additional comments.
